# How remote working increases the importance of positive leadership for employee vigor

**DOI:** 10.3389/fpsyg.2023.1089557

**Published:** 2023-01-20

**Authors:** Marjolein C. J. Caniëls

**Affiliations:** Faculty of Management, Open Universiteit, Heerlen, Netherlands

**Keywords:** positive leadership, well-being, vigor, psychological energy, remote working, turbulent times, COVID-19

## Abstract

**Introduction:**

Leadership is essential for creating a healthy and happy work environment for employees. Due to the COVID-19 pandemic, working remotely from home has become prevalent for many employees, which challenges leaders to reach out to their followers even if these followers are not physically at work. Drawing on positive psychology theories, the aim of this study is to investigate the relationship between positive leadership and psychological energy (i.e., vigor), and particularly the extent in which this relationship is affected by whether employees are working from home, as well as the tenure of the leader-follower relationship.

**Methods:**

A two-wave time-lagged study design is used with a sample of 186 followers.

**Results:**

Findings indicate that the effect of positive leadership on followers’ vigor is especially strong when employees work from home, and even more so when leaders and followers have a long lasting work relationship.

**Discussion:**

The study shows that positive leadership behaviors are positively related to employee vigor. Such positive leadership behaviors consist of praising follower’s individual performance, personally thanking followers, cheering them up, and helping them with specified tasks.

## 1. Introduction

Leadership has been found to be associated to organizationally relevant outcomes, such as employee motivation, work behavior, and performance (Tummers and Bakker, 2021). Also the link between leadership and employee wellbeing has been topic of abundant study (e.g., Gilbreath and Benson, 2004; Kelloway et al., 2012), indicating that leadership is an important predictor of the mental and physical wellbeing of followers (Gilbreath and Benson, 2004). Adopting the argumentation of positive psychology (Seligman and Csikszentmihalyi, 2000), this study will focus on positive leadership. i.e., “behaviors that are enacted by leaders and result in increasing followers’ experience of positive emotions” (Kelloway et al., 2013, p. 108). Positive leadership entails behaviors such as thanking and praising followers and cheering them up. Positive leadership is expected to be especially relevant in turbulent times (Sinclair et al., 2020).

Over the past years, the nature of work has changed. The COVID-19 pandemic caused many people to lose their jobs, change their jobs, or adapt their ways of working to working remotely from home (Kniffin et al., 2021). In these years, remote work has become vastly more accepted for a large variety of jobs. Technical innovations, such as fast and safe internet connections, have provided the opportunity to many employees to schedule meetings *via* video conferencing and working on the office computer from home *via* Virtual Private Network (VPN services). Furthermore, working from home has become more socially accepted at the workplace, as even the most technically challenged and computer-averse people have experimented with working from their home office during the height of the pandemic and have discovered its advantages (Aczel et al., 2021; Ipsen et al., 2021). With the established habit of working remotely on at least some workdays each week, the workplace has changed. Therefore, also leadership has to change to ensure happy, healthy, and high-performing workers (Coun, 2021; Foss, 2021).

A useful indicator of employee wellbeing is vigor (Bakker et al., 2008; Shirom, 2011). Vigor is an important dimension of work engagement. It refers to the extent in which employees feel strong and vigorous when working and attain a work-related positive and fulfilling state of mind (Schaufeli and Bakker, 2004a; Schaufeli and Salanova, 2007). Vigor reflects “individuals’ feelings that they possess physical strength, emotional energy, and cognitive liveliness” (Shirom, 2011, p. 50). Due to the COVID-19 pandemic, working from home has become prevalent for many employees, which provides challenges to leaders, as it may become more difficult for them to reach out to their followers when these followers are not physically present at the workplace. It may be so that employees working from home have more need for positive leadership than employees who are physically present at work, especially during mentally and physically taxing times. Therefore, the question becomes whether and to what extent positive leadership behaviors contribute to vigor (i.e., psychological energy) of employees, and whether this relationship is conditioned by the extent to which employees work from home and by the duration of the leader-follower relationship.

Drawing on positive psychology theories (Seligman and Csikszentmihalyi, 2000), the aim of this study is to investigate the relationship between positive leadership and vigor, and particularly the extent in which this relationship is moderated by a three-way interaction between positive leadership × remote work × tenure of the leader-follower relationship. To this end, a two-wave time-lagged study design is used with a sample of 186 leader-follower dyads from Dutch and Flemish organizations. Findings indicate that the effect of positive leadership on followers’ vigor is especially strong when employees work from home, and even more so when leaders and followers have a long-lasting work relationship.

This study contributes to the literature by exploring the moderating effect of remote work on the relationship between perceived positive leadership behaviors and employee psychological energy. Thereby, it responds to the call for leadership research that takes account of the changing nature of work (Coun, 2021), given turbulent times (Burger et al., 2022). By exploring the relationship between positive leadership and employee vigor, this study answers to the call for empirical studies that address the impact of increased working from home due to COVID-19 (e.g., Kniffin et al., 2021). Furthermore, this study’s theoretical viewpoint and empirical results signify a meaningful contribution to the overall occupational health psychology literature. Occupational health psychology has posed the question of how to create and shape “healthy” organizations, that are characterized by the creation of work environments that encourage employee work-related wellbeing and health over time (Cooper et al., 2001; Nerstad et al., 2020). Given turbulent times with increased levels of remote working, there is a need for more insights about psychosocial work environments that foster wellbeing, and consequently, more study is needed of the antecedents of vigor (Fritz et al., 2011; Shirom, 2011). In practice, the current study could offer guidance to organizations and their leaders with respect to how employees can be enabled to maintain their vigor and energy levels, and thus foster their wellbeing at work.

The remainder of the study is organized as follows. Section 2 provides the theoretical background to the hypotheses by justifying the theoretical basis for direct, moderated, and three-way relationships between the key variables of this study. Section 3 discusses characteristics of the sample as well as the method that was employed to gather the data and techniques that were used to analyze the data. Results of relevant analyses are shown in Section 4. Sections 5–7 discuss the results, provide theoretical and practical implications of the study and address limitations, respectively.

## 2. Theoretical background and hypotheses

### 2.1. Vigor and positive leadership

Various studies have connected vigor to employee-level outcomes, such as increased performance, subjective work capacity, and physical health (Shirom et al., 2008; Halbesleben, 2010; Shirom, 2010). Given that vigor captures positive functioning and wellbeing at work, it is of critical importance to further gain insights about the antecedents of vigor (Shirom, 2010, 2011; Nerstad et al., 2020).

Studies have shown that leadership style is an important predictor of vigor (e.g., Shirom, 2011). With the advent of positive psychology (Seligman and Csikszentmihalyi, 2000) and the study of positive organizational behavior (Luthans, 2002; Wright, 2003) scholars have sought to increase their understanding of workplace wellbeing and work engagement and found evidence of a positive relationship between several leadership styles and vigor. Avolio (1999) showed that transformational leadership leads to energizing emotions among employees. Leaders displaying relationship-building behaviors have been found to induce vigor among their followers, either directly, or through a mediation by interpersonal trust and cohesiveness that are fostered by relationship-building behaviors (Carmeli et al., 2009; Shirom, 2011).

While various leadership styles and behaviors have been associated with wellbeing and work engagement, none of these leadership styles fully capture leader positivity. For example, transformational leadership refers to a leadership style in which leaders inspire and motivate followers to not only achieve their goals, but also to perform beyond expectations in order to address collective organizational values and needs (Bass, 2005). Transformational leaders encourage their followers to strive for excellence, try out new ideas, and challenge the status quo in order to bring about positive change in the organization (Bass, 2005). Different definitions of transformational leadership may place emphasis on different aspects, but all agree that inspiring and supporting followers to achieve success for the organization is at the core of transformational leadership (Bass, 2005; Van Dierendonck et al., 2014). In contrast, positive leadership emphasizes leader behaviors that create positive emotions in employees to benefit followers’ wellbeing (Kelloway et al., 2013). Positive leaders prioritize building relationships and creating a workplace where employees feel valued and supported (Kelloway et al., 2013). In other words, whereas transformational leadership focuses on organizational effectiveness (Van Dierendonck et al., 2014), positive leadership primarily emphasizes followers’ needs and wellbeing.

Positive leadership focuses on creating positive work experiences for employees (Kelloway et al., 2013) and it is associated with positive self-concepts (Hannah et al., 2009) and positive (but not negative) affect toward work (Kelloway et al., 2013) and the organization (Youssef-Morgan and Luthans, 2013). Studies have shown a positive association between positive leadership and employees’ positive emotions (Lilius et al., 2008; Cameron and Plews, 2012). Building on Fredrickson’s (2001) broaden-and-build theory of positive emotions, which suggests that positive affective states generate personal resources that are essential for psychological and physical wellbeing (Fredrickson, 1998), it is likely that positive leadership behaviors, by inducing positivity in followers, are positively associated with follower vigor. This idea is consistent with Affective Events Theory (AET, Weiss and Cropanzano, 1996), which indicates that work events may trigger affective reactions in employees, which, in turn, determine their attitudes and behaviors at work. Positive leadership behaviors are expected to create positive work events for their followers and thereby induce vigor. Therefore:

*Hypothesis 1*: Perceived positive leadership is positively associated with follower vigor.

### 2.2. The moderating effect of remote working

The COVID-19 pandemic forced organizations to develop new work routines, including the facilitation of remote working for employees (Kniffin et al., 2021). In the aftermath of the pandemic, it has become clear that remote work is here to stay (Kniffin et al., 2021; Mckinsey, 2021) and currently organizations are considering how to adapt their work routines to this development (Muzio and Doh, 2021). Against this backdrop, it is expected that remote workers’ vigor (as compared to on-site workers’ vigor) may particularly benefit from positive leadership behaviors. The main reason for this relates to the fact that remote-working employees may be especially prone to social isolation, negative thoughts, and feelings of unhappiness (Ipsen et al., 2021). Remote working has been shown to restrain the possibilities for social and informal exchanges with colleagues (Tremblay and Thomsin, 2012; Boell et al., 2016). Remote work and the increased risk of social isolation have been linked to reduced wellbeing and poor performance (Marshall et al., 2007). Furthermore, social isolation and psychological distress mutually affect each other over time, which may induce a negative spiral (Van Zoonen and Sivunen, 2022), specifically for remote workers.

Positive leadership evokes positive feelings in followers (Lilius et al., 2008; Cameron and Plews, 2012). Positive behaviors of leaders may provide rays of sunlight on a dark day, especially for remote workers. Given their proneness to social isolation and detachment, it is expected that for remote workers (more than for office workers), positive interactions with their leaders show them that they are a valuable contribution to the organization and that they are an appreciated part of a workgroup. Positive leadership is invigorating and it may be exactly what remote workers need to remain happy and healthy while doing their job. This is not to deny that office workers also need to feel valued. Yet, by being in the office, office workers have more opportunities to observe their leaders for behavioral cues and they may therefore have less need for explicit confirmations, such as provided by positive leadership. Hence, it is likely that the positive relationship between positive leadership and vigor is particularly strong for remote workers (as compared to office workers).

*Hypothesis 2*: Remote working moderates the positive relationship between positive leadership and vigor, such that this relationship is strengthened for remote workers.

### 2.3. Leader-follower relationship tenure

Leader-follower relationship tenure reflects the duration of the work relationship between a leader and his/her follower and is often associated with leader–follower relationship quality (Guarana and Barnes, 2017). The strength of the leader-follower exchange relationship increases over time (Guarana and Barnes, 2017). When leader-follower relationship tenure is short, leaders and followers are not familiar with each other, which changes when tenure increases and the social exchange relationship becomes stronger (Dienesch and Liden, 1986; Guarana and Barnes, 2017). Long-lasting work relationships are characterized by mutual trust and understanding (Dienesch and Liden, 1986). Leaders in long-lasting leader-follower relationships have a good knowledge about their followers and what makes them tick. Later in the relationship (i.e., at a higher tenure), leaders and followers have a wide set of observations of each other by which they can evaluate the stability of emotional cues, making it easier for leaders to influence these followers. Therefore, leader-follower relationship tenure is expected to further strengthen the moderating effect of remote work on the positive leadership–vigor relationship.

When leader-follower relationship tenure is high, it is expected that positive leadership relates more strongly positive to vigor for remote workers than for office workers. Remote workers are expected to need extra confirmation that their performance is up to par and that they are fulfilling their leaders’ expectations (because of their otherwise social isolation). The positive leadership behaviors (compliments, thanking, and cheering up) provide such confirmations for them. On-site workers with a long dyad tenure may have less need for such explicit confirmations as they may pick them up from less explicit gestures and eye-contact with their leader on-site.

In contrast, when leader-follower relationship tenure is low, no significant difference is expected to be found between remote workers and office workers with respect to the positive leadership-vigor relationship. The justification being that the duration of the leader-follower relationship has been too short for leaders to be able to evaluate stability of emotional cues of office workers and remote workers alike.

In other words, the effect of positive leadership on followers’ vigor is expected to be especially strong for employees who work from home and have a long-lasting work relationship with their leader. That is, a three-way interaction between positive leadership, remote work, and leader-follower relationship tenure may exist.

*Hypothesis 3*: The relationship between positive leadership and vigor is dependent on the interaction effect between remote working and leader-follower relationship tenure. Specifically, given high leader-follower relationship tenure and high levels of remote working, the relationship between positive leadership and vigor becomes more positive, compared to the relationship for individuals with low levels of remote working.

**Figure 1:**
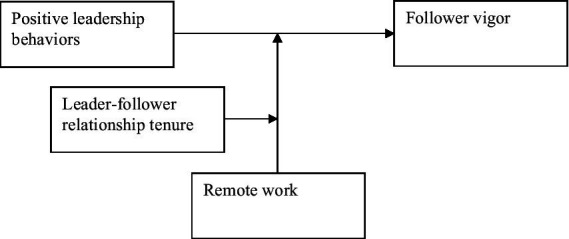
Conceptual model.

Figure 1 summarizes all hypothesized relationships.

## 3. Methods

### 3.1. Sample

Data were gathered in two waves by using the online open-source survey platform LimeSurvey. In the study, 186 employees from various Dutch and Flemish organizations participated. They represented the follower-side in a leader-follower dyad. For the goal of this study, participants filled out two questionnaires, 7 weeks apart. At the first measurement point (T1), data were collected among followers about the perceived leadership behaviors that were shown by their own leader. In this wave also, self-reported data were gathered from employees about whether they have been working from home more than usual due to COVID-19 and its effects on their work. Furthermore, data were collected about the tenure of followers’ work relationship with their leader. Data about the dependent variable, followers’ vigor, were collected at the second measurement point (T2). The Ethics Committee of the researcher’s university approved the study. Informed consent was provided by the participants in the study and a number of procedures were employed to limit common method bias, including pseudonymization, request for honest answers, and the opportunity to stop anytime without the need to provide a reason.

The choice for a 7-week time interval was partly based on established conduct and partly on practical reasons. Other studies capturing behavioral outcomes have used a similar lag (e.g., Cui et al., 2008; Vasey et al., 2014) and have shown that a lag of several weeks is appropriate for extracting information on behavioral patterns (Cui et al., 2008). A 7-week delay between measurements reduces the likelihood that responses given in the first measurement will be remembered and influence responses in the second measurement. There was also a practical reason. The design of the timeline for this study was influenced by the restricted possibilities for respondents of accessing the survey tool.

Two inclusion criteria were employed when selecting respondents for the sample. First, inclusion depended on whether there was a hierarchical leadership relation between the leader and the follower in the dyad. Second, given that the survey was in Dutch, inclusion depended on mastery of the Dutch language.

In total, 246 followers were invited to participate in the study. At the first measurement point, 218 followers completed the questionnaire. After the second wave of data collection, the final sample consisted of 186 followers who filled in the survey on both measurement points (T1 and T2). In this dataset, 57.9% of followers were female. On average, followers were 42.7 years old (*SD* = 12.6) and more than 50% of followers had a bachelor degree or higher.

Respondents were recruited from a multitude of sectors and from organizations of various sizes. For 163 respondents, information about sector and size of the organization was disclosed in the survey. The three most represented sectors are healthcare (27%), financial services (21%), and manufacturing (18%). Most respondents work in large companies with more than 1,000 employees (63%), but also smaller organizations with between 0 and 99 employees are represented in the sample (9%), as well as middle-sized companies (100 to 499 employees: 15%; 500 to 999 employees: 12%).

### 3.2. Measures

Validated scales from prior studies were used to assess the key variables in the current study. Items originating from English scales were translated into Dutch by adopting the back-translation procedure recommended by Brislin (1986).

#### 3.2.1. Positive leadership

At the first measurement (T1), positive leadership was measured by a 5-item scale, validated by Kelloway et al. (2013). Participants were asked to reflect on the past months of work and to indicate how often their supervisor had displayed positive leadership behaviors. An example item is “My leader praised me for my job performance.” Each item was rated on a 5-point scale with higher scores representing a higher frequency of the specific leader behavior. Reliability of the scale was assessed using McDonald’s (1999) omega (ω) in addition to Cronbach’s alpha (α). The reliability analysis showed good internal reliability of the vigor scale (ω = 0.90, α = 0.90).

#### 3.2.2. Working from home

Respondents were asked whether they were working completely from home, or working from home more than usual because of (the aftermath of) COVID-19 (T1). Respondents could answer “yes” (coded “1”) or “no” (coded “2”).

#### 3.2.3. Tenure of the leader-follower relationship

Following Vasquez et al. (2021), leader–follower relationship tenure was measured using the single item “How long have you been working with your current leader?.” Answer categories ranged from “less than one year” (coded “1”) to “more than four years” (coded “5”).

#### 3.2.4. Vigor

At the second measurement (T2), vigor was assessed using the vigor dimension (3 items) of the 9-item Dutch Utrecht Work Engagement Scale (UWES, or UBES in Dutch; Schaufeli and Bakker, 2004b; ω = 0.83, α = 0.83). An example item is “At my work, I feel bursting with energy.” Each item was rated on a 5-point scale, ranging from 1 (never) to 5 (always). Previous studies have extensively used this scale and confirmed its validity and reliability (Schaufeli et al., 2006; Seppälä et al., 2009).

#### 3.2.5. Control variables

Several control variables were assessed, as prior studies have indicated that the demographic background of employees may explain some of the variance in their levels of energy (e.g., Bakker et al., 2005). Respondents were asked to report their year of birth and their gender (coded 0 for male and 1 for female). Education level was evaluated using six levels common to the Dutch and Flemish educational systems (1 = basic education; 2 = high school; 3 = applied education; 4 = higher applied education; 5 = university degree; 6 = PhD).

### 3.3. Analytical strategy

The hypotheses were analyzed using Jamovi open-source software (The Jamovi project, 2022), as well as R Studio (R Core Team, 2021) and various R-packages, including lavaan (Rosseel, 2012) and the Process function for R (Hayes, 2021). Collinearity statistics for the independent variables were calculated. All Variance Inflated Factors (VIFs) were well below the recommended threshold of four (Hair et al., 2017). Model fit was assessed by means of a Confirmatory Factor Analysis (CFA). When all items of the core model variables were included in a four-factor model, the following fit measures were generated: χ2 = 80; df = 31; RMSEA = 0.09; CFI = 0.94; TLI = 0.92, indicating an acceptable fit. The four-factor model fit is preferable over the fit of the one-factor specification of the model (χ2 = 280; df = 35; RMSEA = 0.19; CFI = 0.71; TLI = 0.63). Regression and moderation analyses were used to test the hypotheses. The moderation models were analyzed, using 10,000 bootstrap samples. Following conventional procedures (Aiken et al., 1991), to enhance the interpretability of the analyses, continuous predictor variables were mean-centered prior to constructing the interaction terms for the moderation analyses.

## 4. Results

Table 1 summarizes the means, standard deviations, and correlations between the main variables in the study. All correlations were below the threshold of 0.70 (Tabachnick and Fidell, 2001), indicating that a presence of multicollinearity in the dataset is unlikely. Age and education level are negatively associated with vigor, which is in line with findings from previous studies (Bakker et al., 2005).

**Table 1:** Correlation matrix.

	Mean	SD	1	2	3	4	5	6	7
1. Age	42.7	12.6	—						
2. Gender	0.58	0.5	−0.07	—					
3. Education level	3.92	0.89	−0.40^***^	−0.01	—				
4. Leader–follower relationship tenure (T1)	2.02	0.66	0.37^***^	0.00	−0.23^**^	—			
5. Working from home (T1)	1.6	0.49	−0.19^*^	−0.05	−0.04	−0.07	—		
6. Positive leadership (T1)	3.13	0.75	−0.10	0.02	−0.11	−0.01	0.13	(0.90; 0.90)	
7. Vigor (T2)	3.74	0.65	0.23^**^	−0.06	−0.23^**^	0.04	0.06	0.21^**^	(0.83; 0.83)

SD denotes the standard deviation; (ω; α) reported on the diagonal.

* *p* < 0.05.

** *p* < 0.01.

*** *p* < 0.001.

Table 2 presents the results from the regression and moderation analyses. Model 1 shows the results of the multiple regression of the model variables. It includes the control variables age and education level (*n* = 169), as these controls were flagged in the correlation analysis as having a positive relationship with vigor. The analysis indicates a direct positive association between positive leadership and vigor (*b* = 0.18; *p* = 0.000), thereby supporting Hypothesis 1. Given the limited size of the sample (*n* = 169) and the insignificance of education level as a control variable in Model 1, as well as the small effect size of the correlation between age and vigor in the correlation matrix (*r* = 0.231), it was decided to exclude this control variable in further model specifications, to improve the power of the analyses. Hereby, the underlying study adheres to recommendations of Bernerth and Aguinis (2016) as well as Becker (2005) about parsimonious use of control variables. Including education level as a second control variable reduces the power of the analyses and could limit the possibility of finding significant effects in de moderation analyses (Becker, 2005; Bernerth and Aguinis, 2016). Therefore, the second model (Model 2) only includes age as a control (*n* = 177). The positive direct relationship between positive leadership and vigor is nuanced as soon as the interaction effects are added to the model specification, which are needed to test Hypotheses 2 and 3. Model 2 in Table 2 reports a positive and significant two-way interaction between remote work and positive leadership, which supports Hypothesis 2. Also, a positive and significant three-way interaction was reported (Model 3), which is supportive of Hypothesis 3. Model 4 shows the results of a model without control variables (*n* = 186). Results differ only slightly across models with respect to effect sizes. However, the three-way interaction gains in significance when age is excluded from the model specification (Model 4 vs. Model 3). The pattern of results of the various analyses suggests that Model 3 can be considered the best reflection of the findings, as age has been shown to be a small, though significant, predictor of vigor.

**Table 2:** Results of multiple regression analyses predicting vigor.

	Model 1 linear regression	Model 2 two-way moderation	Model 3 three-way moderation	Model 4 three-way moderation excluding controls
Constant	3.68^***^	3.45^***^	2.96^***^	3.63^***^
Education level	−0.09	−0.08		
Age	0.01^**^	0.01^**^	0.01^***^	
Positive leadership (T1; LPOS)	0.18^***^	−0.30	−0.28	−0.35^*^
Working from home (T1; HOME)	0.08	0.09	0.11	0.06
Leader–follower relationship tenure (T1; LMXT)	−0.06		−0.5^**^	−0.48^**^
LPOS × HOME		0.29^**^	0.29^**^	0.32^***^
LPOS × LMXT			−0.78^***^	−0.80^***^
HOME × LMXT			0.27^*^	0.31^**^
LPOS × HOME × LMXT			0.40^**^	0.43^***^
R^2^	0.12	0.15	0.19	0.13
F Statistic (df = 5; 173)	4.87^***^			
F Statistic (df = 5; 179)		7.78^***^		
F Statistic (df = 8; 186)			6.71^***^	
F Statistic (df = 7; 186)				5.03^***^

* *p* < 0.1.

** *p* < 0.05.

*** *p* < 0.01.

**Figure 2:**
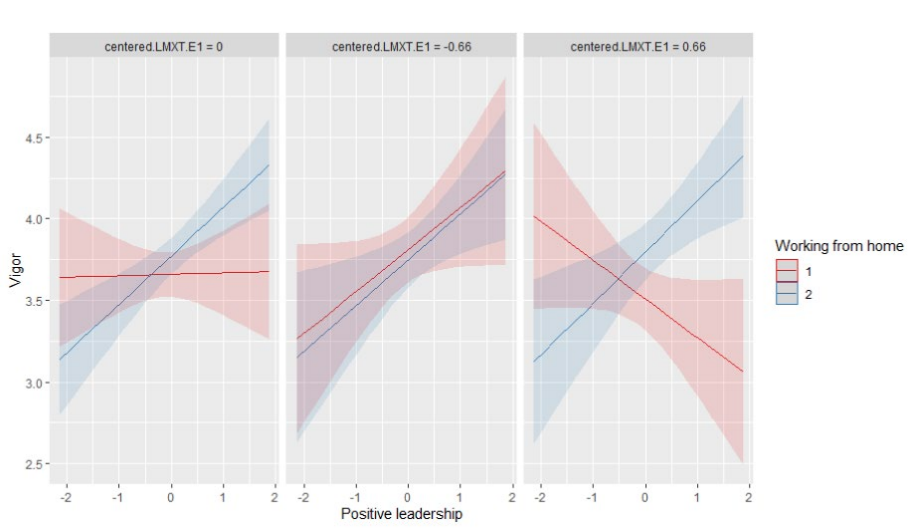
Three-way interaction (positive leadership × working from home × leader-follower relationship tenure) on the relationship between positive leadership behaviors and vigor. LMXT.E1 denotes leader-follower relationship tenure; Working from home “yes” is coded “1,” “no” is coded “2.”

Models 2 and 3 indicate significant two-way and three-way interactions. Given these significant interactions, a simple slope analysis has been performed for Model 3. Simple slopes were tested for low (one standard deviation below the mean), moderate (mean), and high (one standard deviation above the mean) levels of the moderators, as recommended by Aiken et al. (1991). In Figure 2, the three-way interaction was plotted using the R package sjPlot (Gelman, 2008), which provides an illustration of the interaction effects. The left panel in Figure 2 shows the interaction between remote work and positive leadership for an average level of leader-follower relationship tenure. In essence, this panel reflects the two-way interaction between remote work and positive leadership. The middle panel in Figure 2 shows the three-way interaction for low (one standard deviation below the mean) values of leader-follower relationship tenure. The two regression lines all but overlap. However, when looking at the right panel in Figure 2, which reflects the situation at high (one standard deviation above the mean) levels of leader-follower relationship tenure, it can clearly be seen that the regression lines differ for working remotely from home (blue, upward-sloping line) and not working remotely from home (red, downward-sloping line). This finding supports Hypothesis 3, which poses that remote workers experience a stronger positive relationship between positive leadership and vigor than workers who do not work remotely (more than before the pandemic).

## 5. Discussion

While various studies have explored the relationship between leadership and wellbeing of employees at work, the plausible effect of leader positivity has not been captured in previous empirical studies. The current study focused on the relationship between positive leadership behaviors and one important aspect of employee wellbeing, namely vigor. Vigor reflects feelings of psychological energy and physical strength (Shirom, 2011). The results of the present study indicate that positive leadership indeed is positively associated with vigor, and this positive relationship is especially strong for followers who are working remotely from home (more than before the pandemic). Moreover, the moderating effect of remote work is further enhanced for followers who experience a long leader-follower relationship tenure.

The results of this study generate several theoretical and practical implications. First, the present study contributes to studies in the line of positive psychology (Seligman and Csikszentmihalyi, 2000). Specifically, this study contributes to broaden-and-build theory (Fredrickson, 2001), by investigating positive leadership behaviors and their association with employee wellbeing (vigor). Where previous studies have associated employees’ positive affective states with the development of personal resources that are vital for employee wellbeing (Fredrickson, 2001), the present study explicitly provides evidence of positive leadership behaviors and the conditions under which these behaviors are associated with employee wellbeing (i.e., vigor). In this way, the present study extends current knowledge about antecedents of vigor (Fritz et al., 2011; Shirom, 2011). Positive leadership focuses on increasing positive work experiences for employees (Kelloway et al., 2013). This notion is supported by the findings of the current study that indicate that positive leadership behaviors indeed are positively associated with follower vigor (Hypothesis 1).

Second, by exploring conditions under which positive leadership behaviors are associated with vigor, this study responds to recent calls for leadership research that addresses the changing organization of work, which includes the accelerated uptake of flexible work arrangements (Coun, 2021; Foss, 2021), and which may imply an increased focus on individual objectives and rewards (Foss, 2021), thereby intensifying the importance of the leader-follower relationship (Spicer, 2020). The present study shows that specifically remote workers’ vigor (as compared to on-site workers’ vigor) benefits from positive leadership behaviors. The positive events generated by leaders are especially advantageous for followers who are less in the office than before the pandemic and may be susceptible to negative and depressive thoughts induced by their social isolation from work. To that account, the left panel in Figure 2 shows the situation under average levels of dyad tenure. As evidenced by the red horizontal line, positive leadership behavior does not seem to affect follower vigor of employees who have not increased the amount of working from home due to the pandemic. Only remote workers experience a significant positive association, as evidenced by the blue upward-sloping line (supportive of Hypothesis 2). Notably, the underlying study did not include information about the actual number of days that participants were working from home prior to the pandemic. If participants prior to the pandemic were not working from home at all, the pandemic may have instigated an especially intense adaptation process. The positive events generated by leaders may be particularly advantageous for followers who underwent such an intense adaptation (from 5 days in the office to remote working) and who may be especially susceptible to negative and depressive thoughts induced by their (sudden) social isolation from work. Future research is needed to explore whether empirical evidence of such an effect can be found.

Analyses for Hypothesis 3 indicate that the situation is even more nuanced than was thought on basis of the results found with respect to Hypothesis 2. Figure 2 shows the importance of taking into account the leader-follower relationship tenure (Hypothesis 3). The middle panel reveals that under low leader-follower relationship tenure, the relationship between positive leadership and vigor is equally strong and positive for remote workers and on-site workers. Though, under high leader-follower relationship tenure (right panel in Figure 2), a clear difference becomes visible between remote and on-site workers (upward-sloping blue line vs. downward-sloping red line). This pattern of results can be interpreted as follows. At the early stages of the leader-follower relationship, positive leadership behaviors help increase the vigor of all employees (working remotely or on-site) as evidenced by the middle panel in Figure 2. Followers with long-lasting work relationships with their leader only profit from their leader’s positive behaviors when working remotely (right panel in Figure 2, upward-sloping blue line). Apparently, remote workers need confirmation that their performance is up to par and they are fulfilling their leaders’ expectations. The positive leadership behaviors (compliments, thanking, cheering up) provide such confirmations for them. On-site workers (with a long dyad-tenure, signified by the downward-sloping red line in the right panel of Figure 2) may have less need for such explicit confirmations as they may pick them up from less explicit gestures and eye-contact with their leader on-site. This reasoning is consistent with social learning theory (Bandura, 1997; Walumbwa et al., 2010), which states that followers often employ observational learning, i.e., followers observe their leaders for behavioral cues about what is expected from them. It is likely that remote workers have only a limited opportunity to observe these cues while working remotely. Explicit positive leadership behaviors fill this need and thereby induce vigor in remote workers with a long relationship tenure. The difference with regard to on-site workers (red upward-sloping line vs. red downward-sloping line in the middle and right panel of Figure 2) can be similarly explained. On-site workers with a long-standing work relationship with their leader (right panel, downward-sloping red line) can more easily evaluate their leaders implicit cues than on-site workers with a short dyad tenure (middle panel, upward-sloping red line), making it easier for them to interpret these implicit cues. The explicit cues provided by positive leadership behaviors may be felt as superfluous and even irritating, thereby depleting their resources (vigor) instead of feeding them. Supporting this notion are studies that have shown that too much of a good thing can be bad (Pierce and Aguinis, 2013; Busse et al., 2016).

## 6. Practical implications

The current study’s findings provide insights and practical guidelines to organizations and their leaders regarding the question as to how employees can be supported to enhance their vigor and energy levels, thereby supporting their wellbeing at work. The study provides evidence showing that positive leadership behaviors are positively related to employee vigor. Such positive leadership behaviors consist of praising followers, individual performance, personally thanking followers, cheering them up, and helping them with specified tasks. All of these leader behaviors are concrete and easy to operationalize in a work-setting. Human resource management departments could organize training sessions for leaders targeted at further developing their positive leadership behaviors.

The development of relationship-building behaviors may also be included in such trainings, as the present study indicated the positive working of leader-follower relationship tenure. Dyad tenure in itself is not easy to influence, but relationship-building skills are expected to positively affect the leader-follower dyadic relationship (Palanski and Yammarino, 2011) and such skills have been shown to be positively related to vigor (Carmeli et al., 2009).

This study’s findings show that especially workers who are working remotely (more than before) are benefiting from positive leadership. Organizations could offer their group of remote workers possibilities for engaging with a vitaly coach, who specifically provides emotional support and fills the gap that potentially is left by a leader. Such interactions focused on coping and increasing personal resources are likely to alleviate stress during remote work and has been found to enhance psychological wellbeing (Kaslow et al., 2020).

## 7. Limitations

Some limitations need to be acknowledged. Firstly, although transformational leadership behaviors have been shown to conceptually differ from positive leadership behaviors, Kelloway et al. (2013) have suggested that positive leadership may act as a partial substitute for transformational leadership behaviors in cases when transformational leadership behaviors are absent. Future studies may want to include transformational leadership behaviors as well as positive leadership behaviors to explore their joint effect on follower vigor. Relatedly, the data used for this study did not provide information about social relationships of employees next to their work relationship with their leader. It may be the case that some employees experienced a boost (or drop) in their (workplace) vigor because of their social relationships with colleagues or friends and family. Social relationships with others is a relevant construct to consider in future research, as social relationships may also explain part of the variance in employee vigor. Furthermore, while the present study provides evidence of the positive relationship between positive leadership and vigor, this is not to deny that leadership behaviors aimed at increasing followers’ vigor could potentially have drawbacks for employees. Leaders who know how to increase their followers’ vigor could potentially misuse these abilities and as an effect disengage and frustrate their followers (Nikolova et al., 2021). The present study did not investigate whether positive leadership can also have such negative effects on employees, for example in terms of increasing burnout. Future studies may want to include not only measures of positive work outcomes (vigor, wellbeing), but also negative work outcomes (frustration, disengagement, and burnout) to check for possible downsides of positive leadership.

Secondly, by studying the conditions under which positive leadership is related to follower vigor, it is implicitly assumed that certain leader behaviors are instrumental to follower behavior. Alternative leadership approaches, such as authentic leadership, advocate that leaders influence followers through positive modeling (Adams et al., 2020), which may increase followers’ vigor as well. Authentic leaders are self-aware, understand their own strengths and weaknesses, and are able to regulate their behavior to reflect their own norms and values (Avolio and Gardner, 2005; Ilies et al., 2005; Adams et al., 2020). Studies in this line focus on personal qualities and characteristics of leaders (Adams et al., 2020), which may or may not manifest in positive leader behaviors and which, in turn, may or may not be mirrored by followers. In the present study, a more direct approach is chosen that explicitly focuses on how leaders relate to their followers, in terms of praising and thanking them (Kelloway et al., 2013). Future studies may want to investigate trickle-down effects (Masterson, 2001; Wo et al., 2019) and leader-follower interdependence models as a way to gain insight into alternative ways to influence followers’ vigor.

Thirdly, with regard to the positive leadership scale, the present study slightly adapted the original phrasing of Kelloway et al. (2013), who asked respondents to reflect on the past 4 months, while in the underlying study, participants were asked to reflect on the past months of work. It would have been preferable to have indicated a specific number of months, although arguably results from a reflection by participants on the past months may not differ much from a reflection on the past 4 months. Nevertheless, future studies are advised to denote a specific number of months while adopting the positive leadership scale.

Finally, this study incorporated multiple waves of data gathering. Consequently, the final sample size is quite limited, which may have affected the power of the analyses and the accuracy of the estimates (Shadish et al., 2002). Nevertheless, a multi-wave study design allows for more rigorous testing of hypotheses than a cross-sectional study design. By having a 7-week time lag between evaluating the dependent and independent variables, the study adheres to Podsakoff et al.’s (2003) recommendations for reducing various method biases through a time-lagged study design. Further studies into positive leadership behaviors and vigor are advised to pursue large multi-wave samples in order to overcome power issues.

These limitations aside, the present study’s findings have advanced current understandings about whether and under what conditions positive leadership behaviors are associated with employee psychological energy, i.e., vigor.

## Data availability statement

The datasets presented in this article are not readily available because respondents of the survey have not consented to sharing the data. Requests to access the datasets should be directed to MC, marjolein.caniels@ou.nl.

## Ethics statement

The studies involving human participants were reviewed and approved by Research Ethics Committee (cETO) of the Open Universiteit (Netherlands). The patients/participants provided their written informed consent to participate in this study.

## Author contributions

MC gathered the data, performed the analyses, and wrote the paper.

## Conflict of interest

The author declares that the research was conducted in the absence of any commercial or financial relationships that could be construed as a potential conflict of interest.

## Publisher’s note

All claims expressed in this article are solely those of the authors and do not necessarily represent those of their affiliated organizations, or those of the publisher, the editors and the reviewers. Any product that may be evaluated in this article, or claim that may be made by its manufacturer, is not guaranteed or endorsed by the publisher.
